# *In vivo* assessment of arterial stiffness in the isoflurane anesthetized spontaneously hypertensive rat

**DOI:** 10.1186/1476-7120-12-37

**Published:** 2014-09-17

**Authors:** Eric E Morgan, Andrew B Casabianca, Samer J Khouri, Andrea L Nestor Kalinoski

**Affiliations:** Department of Physiology and Pharmacology, University of Toledo, College of Medicine and Life Sciences, 3000 Arlington Ave, Toledo, OH 43614 USA; Department of Anesthesiology, University of Toledo, College of Medicine and Life Sciences, 3000 Arlington Ave, Toledo, OH 43614 USA; Department of Medicine, University of Toledo, College of Medicine and Life Sciences, 3000 Arlington Ave, Toledo, OH 43614 USA; Department of Surgery, University of Toledo, College of Medicine and Life Sciences, 3000 Arlington Ave, Toledo, OH 43614 USA; The University of Toledo Advanced Microscopy & Imaging Center, University of Toledo, College of Medicine and Life Sciences, 3000 Arlington Ave, Toledo, OH 43614 USA

**Keywords:** Blood pressure, Arterial stiffness, Pulse wave velocity, Arterial elasticity index, Doppler, Ultrasound, Isoflurane, Anesthesia, SHR, Rat

## Abstract

**Background:**

Rodent models are increasingly used to study the development and progression of arterial stiffness. Both the non-invasive Doppler derived Pulse Wave Velocity (PWV) and the invasively determined arterial elastance index (EaI) have been used to assess arterial stiffness in rats and mice, but the need for anesthetic agents to make these *in vivo* estimates may limit their utility. Thus, we sought to determine: 1) if known differences in arterial stiffness in spontaneously hypertensive rats (SHR) are detectable by PWV and EaI measurements when made under isoflurane anesthesia, and 2) if these two uniquely acquired assessments of arterial elasticity correlate.

**Methods:**

We obtained PWV and EaI measurements in isoflurane anesthetized young and old SHRs, which are known to have significant differences in arterial stiffness. Doppler pulse waves were recorded from carotid and iliac arteries and the distance (D) between probe applantation sites was recorded. Simultaneously, an EKG was obtained, and the time intervals between the R-wave of the EKG to the foot of the Doppler waveforms were measured and averaged over three cardiac cycles. Pulse-transit time (T) of the carotid to iliac artery was determined, and PWV was calculated as Distance (D)/Time (T), where D = the distance from the carotid to the iliac notch and T = (R to iliac foot) - (R to carotid foot). EaI was subsequently determined from pressure volumes loops obtained via left ventricle catheterization.

**Results:**

PWV and EaI were found to be significantly faster in the older rats (13.2 ± 2.0 vs. 8.0 ± 0.8 m/sec, p < 0.001; 120 ± 20 vs. 97 ± 16 mmHg/μl/g, p <0.05). Bland-Altman analyses of intra- and inter-observer measures demonstrate a statistically significant relationship between readings (p < 0.0001). PWV and EaI measurements were found to be significantly and positively correlated with a correlation coefficient of 0.53 (p < 0.05).

**Conclusion:**

Our study suggests that isoflurane administration does not limit Doppler PWV or EaI measures in their ability to provide accurate, *in vivo* assessments of relative arterial stiffness in isoflurane anesthetised SHR rats. Furthermore, PWV data obtained in these rats correlate well with invasively determined EaI.

## Introduction

Decreased arterial elasticity, which results in increased stiffness of the central elastic arteries such as the aorta, is the primary cause of increased systolic pressure with advancing age and is a powerful independent predictor of cardiovascular disease, mortality and morbidity [1–3]. The processes by which arterial stiffness increases with age, however, are not well understood, and there is a growing interest in identifying and understanding the genetic components and mechanisms that regulate this phenomenon. Rodent models are increasingly being utilized to study the development and progression of arterial stiffening, and both the non-invasive Doppler derived pulse wave velocity (PWV) and the invasively determined effective arterial elastance index (EaI) have been used to assess arterial stiffness in mice and rats [4–13].

PWV is classically determined by assessing the difference in propagation of arterial pressure waves between two recording sites in the line of pulse travel and calculating the delay between corresponding points on the wave (of pressure or flow). PWV has been shown to be closely related to the degree of intrinsic elasticity of the arterial wall [14] and has come to be regarded as the “gold standard” measure of arterial stiffness [15]. Clinically, PWV is most often determined by arterial tonometry, which uses pressure sensors to detect carotid and femoral pulses and the electrocardiogram (EKG) as a timing reference to determine the time delay or “transit time” between the foot of carotid and femoral pulse waveforms. More recent studies have shown, however, that PWV can be accurately determined from the transit time between 2-D guided Doppler flow signals in the carotid and femoral arteries [16] and that these Doppler derived measures compare well to those obtained via arterial tonometry [17]. In a similar manner, the Doppler derived PWV has been obtained in rats and mice utilizing a variety of anesthetics and custom made or specialized EKG triggered instrumentation developed for this purpose [4–8].

Another index of arterial stiffness, the effective arterial elastance (Ea), was first proposed by Sunagawa et al. in 1983 [18] and is an invasively determined parameter of arterial load that is inversely related to arterial compliance [19, 20]. Ea is determined from pressure-volume loops as the negative slope of the line joining the end-diastolic volume and end-systolic pressure points. Although many factors can affect Ea, age associated increases have been well described, and these increases are largely attributed to increased arterial stiffness [21]. Ea can be obtained in small animals from commercially available pressure-volume conductance catheters, but this method has the considerable disadvantage of being a terminal procedure, and thus is unsuitable for longitudinal studies.

The need to give anesthetic agents in order to make these *in vivo* assessments of arterial stiffness may limit the utility of these measures and confound the results of the studies. Indeed, a previous study conducted by Yu et al. that utilized aortic ring preparations from normotensive Wistar-Kyoto rats and SHRs showed that volatile anesthetics may have different relaxing effects on the vasculature of SHR rats compared to their normotensive counterparts [22]. It is not entirely clear, however, what influence isoflurane has on *in vivo* measurements of PWV and EaI measurements in SHR rats. Thus, the goals of this study were 1) to determine if known differences in arterial stiffness could be detected by PWV using conventional ultrasound equipment and by EaI measurements made in SHR isoflurane anesthetized rats, and 2) to determine if the non-invasively obtained Doppler PWV assessment of arterial elasticity correlates with the invasively determined EaI obtained under isoflurane anesthesia. To achieve these goals, we acquired PWV and EaI in isoflurane anesthetized young and old rats, which are well known to have significant differences in arterial stiffness [23–25].

## Methods

### Study design

This study was conducted in accordance with the Guide for the Care and Use of Laboratory animals, published by the National Institutes of Health (NIH Publications No. 85–23) and approved by the Institutional Animal Care and Use Committee at The University of Toledo College of Medicine and Life Sciences. Inbred spontaneously hypertensive rats (SHR/NHsd) obtained from Harlan (Indianapolis, IN) were used to establish colonies that were maintained in the Division of Laboratory Animal Resources at The University of Toledo Health Science Campus (UTHSC) and will be referred to as SHR. The rats were housed 2–3 per cage on a 12:12 hour light–dark cycle with the light cycle coinciding with daytime, and standard rat chow and water were provided *ad libitum*. Ten 12 week old (young) male SHRs weighing ~290 g and ten 32 week old (old) male SHRs weighing ~390 g underwent ultrasound evaluation of carotid and iliac arterial flow using color-guided Doppler pulse wave analysis for determination of PWV (described in detail below). The same rats subsequently underwent cardiac catheterization for invasive determination of effective arterial elastance (see below). All ultrasound and cardiac loop data were subsequently analyzed off-line in an investigator blinded fashion.

### Doppler ultrasound measurement of pulse wave velocity

**Figure 1:**
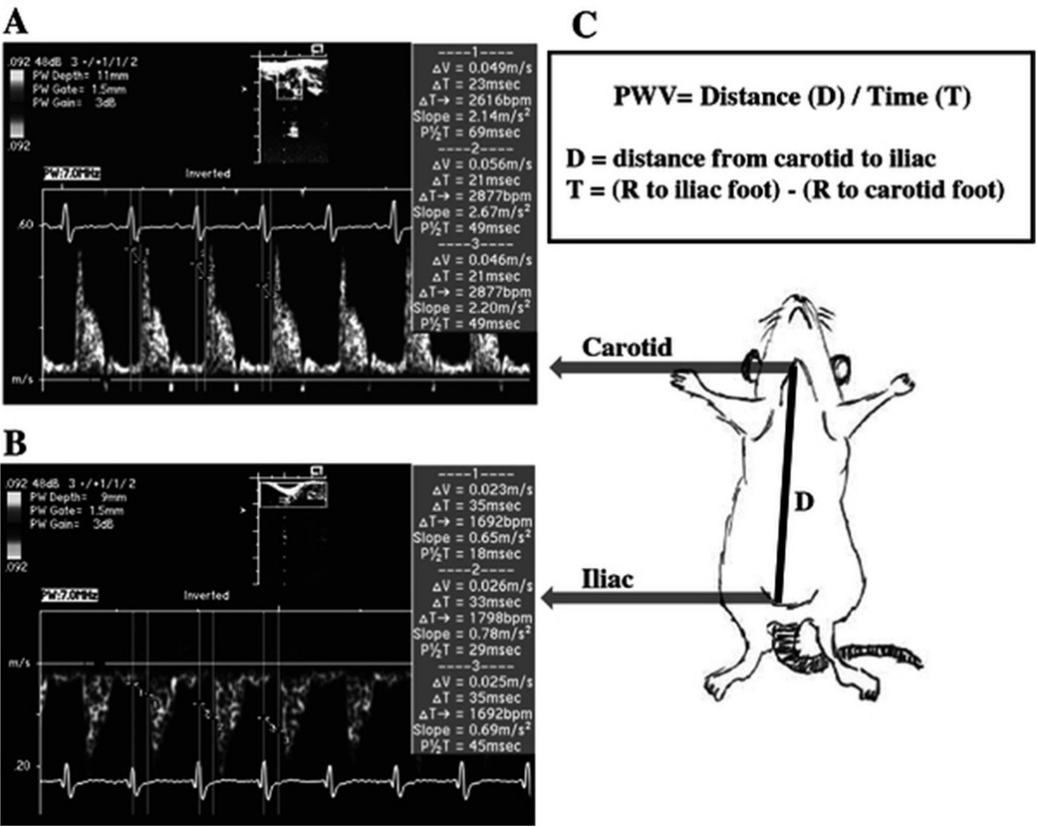
**Calculation of PWV.** Time (T) between the R wave of the EKG and the foot of the **A)** carotid and **B)** iliac pulse waves were determined and averaged over 3 consecutive beats. **C)** PWV was calculated as Distance (D)/ Time (T), where D = the distance from the carotid to the iliac applantation sites and T = the time (R to iliac foot) - (R to carotid foot).

To determine PWV, rats were anesthetized in an induction chamber with 2-3% isoflurane, and a level of anesthesia sufficient for acquiring ultrasound data was maintained with 1.0-1.5% isoflurane (delivered by 100% O_2_ mask inhalation). The neck and inner right thigh were shaved and de-haired with a depilatory cream. The animal was situated in the supine position on a controlled heating pad to maintain a body temperature of 37°C and EKG limb electrodes were placed. An Acuson Sequoia C512 Ultrasound System with a 15 MHz linear array transducer and color-flow Doppler capabilities (Siemens Medical) was used to scan the carotid and iliac arteries. A small amount of echo gel was applied to the neck (for carotid measurement) and to the right thigh (for iliac measurement) in order to improve the conductance of the ultrasound probe. Color-flow Doppler was employed to help locate the arteries and guide placement of the sample gate for obtaining pulse wave forms. The probe was directed parallel to blood flow, and the angle was maintained at less than 20 degrees. The pulse wave sample gate was made as small as possible and its position adjusted to obtain the clearest pulse wave forms. EKG and Doppler signals were then recorded simultaneously at a sweep speed of 200 mm/sec for several cardiac cycles, and the data were stored for subsequent off-line analysis. The total time for each study was approximately 10 minutes. At the end of the study, the distance measured in mm (D) between the points of probe applanation over the carotid and iliac arteries was measured using a tape measure (Figure 1). The time intervals (measured in msec) between the R-wave of the EKG to the foot of the Doppler carotid and iliac waveforms were averaged over three cardiac cycles (Figure 1), and the pulse-transit time from the carotid to iliac arteries (T) was calculated by subtracting the mean R-carotid foot time interval from the mean R-iliac foot time interval. PWV was then calculated as: PWV = Distance (D)/Time (T), where D is the distance in mm from carotid to iliac applanation sites and T = (R to iliac foot) - (R to carotid foot) in msec.

To assess intra- and inter-observer variability of wave propagation time, the intervals between the R-wave of the EKG to the foot of the Doppler carotid and iliac waveforms were re-measured in a randomly selected subset of animals (n = 5 per group) by two independent observers under the same conditions. One scorer analyzed the data twice on separate days to evaluate intra-observer differences. Intra- and inter-observer variabilities were calculated as the absolute value of the differences between the two observations divided by the average of the two observations. To asses repeatability, accuracy and intra-operator consistency in probe placement, angling and path measurement, we repeated the PWV assessment in a second group of older SHR animals (n = 14) and compared the two data sets.

### Measurement of systolic blood pressure

To help evaluate the influence of blood pressure on PWV, systolic blood pressure (SBP) measurements were obtained from a separate group of 9 male SHRs at the younger (12 weeks) and older (32 weeks) ages. Tail cuff measurements were acquired as previously described [26]. Briefly, conscious, restrained rats were warmed to 28 degrees C, and SBP was measured on each rat for 2 consecutive days by two separate operators and averaged for the 12 and 32 week time points.

### Invasive measurement of arterial elastance

For determination of effective arterial elastance (Ea), rats were anesthetized in an induction chamber with 2-3% isoflurane, and anesthesia was maintained so that toe-pinch, tail-pinch and palpebral reflexes were absent (2-3% isoflurane delivered by 100% O_2_ mask inhalation). Rats were placed on a controlled heating pad to maintain a body temperature of 37°C, and a 2.0 F microtip pressure-volume catheter (SPR-838; Millar Instruments; Houston, TX) was introduced via the right carotid artery into the left ventricle (LV) as previously described [27]. After a 20 minute stabilization period, LV pressure-volume loops were recorded using the MPVS 400 pressure-volume conductance system (Millar Instruments) at a sampling rate of 1000/sec for 30 sec. 15–20 pressure-volume loops were subsequently selected and analysed using a cardiac pressure-volume analysis program (PVAN 3.5; Millar Instruments) as previously described [27]. Ea was calculated as the ratio of end systolic pressure to stroke volume and normalized to body weight resulting in the arterial elasticity index (EaI) [10].

### Statistical analysis

Data are presented as mean ± SD. The groups were compared with Student’s *t*-test. PWV was correlated with EaI using the Pearson product moment correlation, as data were normally distributed. The agreements between inter- and intra-observer measurements were tested by Bland-Altman analysis. Differences were considered significant when P < 0.05.

## Results

### Pulse wave velocity

PWV was successfully determined in all 20 rats. The pulse-transit time and path length were found to be significantly different between the two groups, with the younger rats exhibiting a shorter path length and longer transit time (Table 1). The mean PWV in the young rats was 8.0 ± 0.8 m/sec (range 6.3 to 9.3 m/sec). The mean PWV in the older rats was 13.2 ± 2.0 m/sec (range 10.0 to 16.8 m/sec). The calculated PWV was found to be significantly higher in the older rats compared to the younger group (Table 1, p < 0.001). Heart rates were not different between the two groups (439 ± 34 vs. 432 ± 32, p = 0.62, Table 1).

**Table 1:** **PWV parameters assessed by** ***in vivo*** **Doppler ultrasound**

Age	n	Heart rate (bpm)	Distance (D) (mm)	R to Carotid foot (msec)	R to Iliac foot (msec)	Time (T) (msec)	PWV (m/sec)
SHR (young)	10	439 ± 34	134.0 ± 9.7	24.3 ± 2.9	41.1 ± 2.3	16.8 ± 1.3	8.0 ± 0.8
SHR (old)	10	432 ± 32	156.0 ± 10.7	23.8 ± 3.9	35.9 ± 3.8	12.1 ± 2.3	13.2 ± 2
		N.S.	*P* < 0.001	N.S.	*P* < 0.01	*P* < 0.001	*P* < 0.001

D = distance from the carotid to the iliac notch.

R = R wave of the EKG.

T = (R to Iliac foot) – (R to Carotid foot).

Data are presented as mean ± SD.

### Intra- and inter-observer error

**Figure 2:**
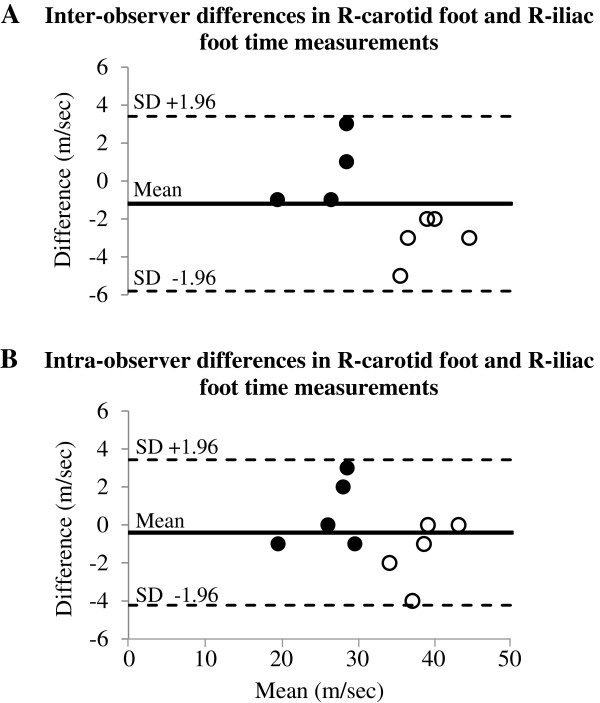
**Bland Altman plot for the percentage of the mean difference and 95% limits for A) Inter- (mean: -1.20%, 95% limits of agreement: 3.40% to -5.80%) and B) Intra- (mean: -0.40%, 95% limits of agreement: 3.43% to -4.23%) observer measurements of R-carotid foot (solid circles) and R- iliac foot (open circles) time measurements.**

Intra- and inter-observer differences in the time determination between the R-wave of the EKG and the foot of the carotid and iliac pulse waves were 5.3 ± 3.0% and 5.2 ± 3.9% for the carotid artery and 5.0 ± 2.5% and 3.9 ± 4.6% for the iliac artery, respectively. These data were found to be positively and significantly correlated, with high R^2^ values for both inter- and intra-observer errors of .97 and .93, respectively. Further, Bland-Altman analysis of these measures demonstrated a statistically significant relationship between readings (p < 0.0001) with very good agreement (Figure 2). Assessment of PWV in the second independent group of older SHR animals revealed that the mean PWV was not different from the first group (12.8 ± 4.4 vs. 13.2 ± 2.0 m/s, p = 0.77).

### Blood pressure

Tail cuff measurements made in a separate group of SHR’s at the younger and older ages revealed no differences in systolic blood pressure at these two time points (189 ± 21 vs. 198 ± 15 mmHg; p = 0.34), respectively.

### Arterial elastance index

**Figure 3:**
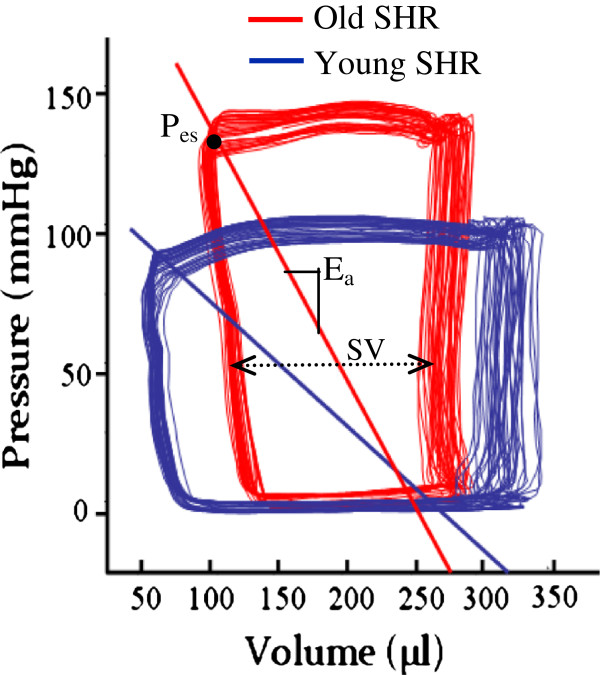
**Representative pressure-volume loops recorded with the Millar pressure-volume catheter system in a young and old rat.** Effective arterial elastance, E_a_ = (P_es_/SV) for each pressure-volume loop series. P_es_ = end systolic pressure and SV = stroke volume.

High quality pressure volume loops were successfully obtained in 8 of the 10 younger and in 7 of the 10 older rats. Figure 3 shows representative pressure-volume loops for both a younger (blue) and older (red) animal. Ea was determined from these pressure-volume loops as the negative slope of the line joining the end-diastolic volume and end-systolic pressure points (Figure 3). Because the older rats were significantly heavier than the younger rats (388 ± 26 vs. 289 ± 9, p < 0.001, Table 2), Ea was normalized to body weight, resulting in the arterial elastance index (EaI). The mean EaI in the younger rats was 97 ± 16 mmHg/μl/g and the values ranged between 75 and 122 mmHg/μl/g. The mean EaI in the older rats was 120 ± 20 mmHg/μl/g and the values ranged between 95 and 144 mmHg/μl/g. EaI was found to be significantly higher in the older rats compared to the younger rats (Table 2, p < 0.05). Heart rates were not different between the young and old rats (346 ± 29 vs. 328 ± 38; p = 0.32).

**Table 2:** **Comparison of Doppler flow measured PWV and calculated EaI by pressure-volume loop analysis**

			Doppler ultrasound		Pressure-volume loops	
Age	n	Weight (g)	Heart rate (bpm)	PWV (m/sec)	Heart rate (bpm)	EaI (mmHg/μl/g)
SHR (young)	10/8	289 ± 9	439 ± 34	8.0 ± 0.8	346 ± 29*	97 ± 16
SHR (old)	10/7	388 ± 26	432 ± 32	13.2 ± 2	328 ± 38*	120 ± 20
		*P* < 0.001	NS	*P* < 0.001	NS	*P* < 0.05

Data are presented as mean ± SD.

*P <0.0001 vs. heart rate measured by ultrasound.

### PWV and EaI correlation

**Figure 4:**
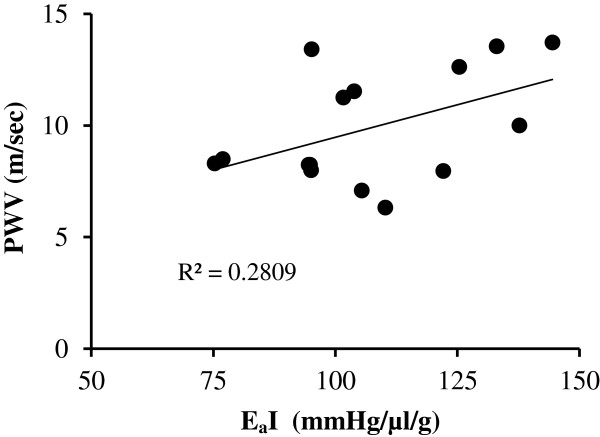
**Pearson correlation analysis of PWV and EaI.**

The PWV and EaI data were found to be positively and significantly correlated, with a Pearson correlation coefficient of 0.53 (Figure 4, p < 0.05).

## Discussion

Pulse wave velocity (PWV) has long been used as a predictor of cardiovascular disease, and an increase in PWV is associated with advancing age and increased morbidity and mortality [28, 29]. The velocity of the pulse wave directly reflects the amount of elasticity in the vascular tree, with a higher PWV indicating increased arterial stiffness [13, 30, 31]. The spontaneously hypertensive rat is a well-characterized model of arterial hypertension that closely mirrors many of the features observed in hypertensive patients and has been used to better understand the genetic components of insulin resistance, neointimal hyperplasia, coronary microvascular remodeling and age related differences in arterial stiffness, among others [13, 23–25, 32, 33]. Although a previous study conducted by Yu et al. suggests that isoflurane may have different effects on SHR vasculature compared to normotensive controls [22], we found that differences in PWV remain detectable between young and old rats under isoflurane anesthesia. As predicted, PWV was significantly faster in the older rats compared to the younger, owing to the increased arterial stiffness observed in these animals. We also found that the use of color-flow Doppler allowed for easy detection of the target arteries and optimal placement of the probe and sample gate for obtaining clear pulse wave forms for subsequent analysis. In fact, the high quality pulse waves obtained with color-flow guidance allowed for easily reproducible calculation of the time intervals between the R-wave of the EKG and the foot of the carotid and iliac pulse waves, with very low inter- and intra-observer errors (Figure 2). Furthermore, our repeatability study demonstrates accuracy and intra-operator consistency in obtaining PWV, suggesting that color-flow Doppler guidance minimizes probe placement, angling, and path measurement errors. Thus, we conclude that color-flow Doppler is very helpful in acquiring high quality pulse waves that allow for reproducible calculation of PWV, and that measurement of Doppler derived PWV provides an effective assessment of relative arterial stiffness in isoflurane anesthetized SHR rats.

We also hypothesized that the effective arterial elastance (EaI), as measured by pressure-volume loop analysis, would be greater in the older SHR rats, and we sought to determine if the non-invasively obtained Doppler PWV estimate of arterial elasticity correlates with the invasive measurements of EaI made under isoflurane anesthesia. Although we were not successful at obtaining EaI in all the animals, due, in part, to technical difficulties encountered in the catheterization procedure and in obtaining pressure-volume loops of sufficient quality for analysis, we were able to demonstrate a significant increase in EaI in the older rats as compared to the younger ones, as well as a significant, positive correlation between EaI and the PWV estimate of arterial stiffness (Table 2, Figure 4).

### Study limitations

Two important limitations of this study merit mention. First, the heart rates determined via cannulation were significantly lower than those obtained during ultrasound assessment for both the younger and older animals (Table 2), owing to the deeper level of anesthesia required for cardiac catheterization. This difference is important as Ea is directly related to heart rate and peripheral resistance [19], both of which can be affected by anesthesia. But rather than keeping the depth of anesthesia (and thus heart rates) the same between the two groups, we chose to conduct the study in a manner which would best reflect the methodologies most likely to be used by others; i.e., administering a lower concentration of isoflurane for determination of PWV than would be used for cardiac cannulation. Given the relatively challenging and terminal nature of obtaining EaI, we felt it worthwhile to report our modest, yet statistically significant correlation between EaI and PWV, despite the observed differences in heart rates.

A second limitation to this study lies in the fact that we used a single rat strain with known differences in arterial stiffness to test our hypothesis that Doppler PWV and EaI measurements can be used to detect differences in arterial elasticity in isoflurane anesthetized hypertensive rats. Although we were successful in this goal, it remains unclear if differences in arterial elasticity caused by other physiologic or pathologic processes or in other rat strains could be as easily detected using either method. It is also important to remember that PWV is influenced by blood pressure. In this study, we found (as have others, [23]) that the blood pressure changes little in the SHR beyond 12 weeks of age. Since the blood pressure is unchanged between the two time points at which PWV was determined, we are able to conclude that the observed differences in PWV are attributable to changes in arterial elasticity and are independent of blood pressure. PWV studies conducted in animal models that exhibit simultaneous changes in arterial stiffness and blood pressure, however, will require blood pressure normalization. Thus, additional studies using other rat strains or models employing pharmacological agents to alter arterial compliance and blood pressure, as has been done by others [10, 34] may further help to define the role of Doppler derived PWV and EaI in assessing arterial elasticity in the hypertensive isoflurane anesthetized rat.

## Conclusion

The present study demonstrates that Doppler derived pulse wave velocities obtained from isoflurane anesthetized spontaneously hypertensive rats can be used to provide a reproducible, non-invasive, *in vivo* assessment of arterial stiffness. Furthermore, our work shows that PWV data obtained in the anesthetized rats correlate with invasively determined EaI. These findings are of interest insofar as longitudinal studies aimed at examining the development and progression of arterial stiffening in hypertensive rats may benefit from the application of a non-invasive, repeatable *in vivo* assessment of arterial elasticity that is valid under isoflurane anesthesia.

## Abbreviations

PWV: Pulse wave velocity
EaI: Arterial elastance index
SHR: Spontaneously Hypertensive Rat
D: Distance from the carotid to iliac artery applantation sites; T = (R to iliac foot) - (R to carotid foot)
P_es_: End systolic pressure
SV: Stroke volume.
